# Diagnostic and Therapeutic Particularities of Sepsis in Hemodialysis Patients

**DOI:** 10.3390/life15091488

**Published:** 2025-09-22

**Authors:** Maria-Daniela Tanasescu, Andrei-Mihnea Rosu, Alexandru Minca, Andreea-Liana Rosu, Maria-Mihaela Grigorie, Delia Timofte, Dorin Ionescu

**Affiliations:** 1 Department of Semiology—Emergency University Hospital, Carol Davila University of Medicine and Pharmacy, 022328 Bucharest, Romania; maria.tanasescu@umfcd.ro (M.-D.T.); alexandru.minca@umfcd.ro (A.M.); dorin.ionescu@umfcd.ro (D.I.); 2 Department of Cardiology, Prof. Dr. Agrippa Ionescu Emergency Hospital, 077015 Balotesti, Romania; 3 Department of Clinical Pharmacology, BBraun, 013714 Bucharest, Romania; andreea.rosu@bbraun.com; 4 Department of Dentistry, Discipline of Endodontics, Faculty of Dentistry, Carol Davila University of Medicine and Pharmacy, 020021 Bucharest, Romania; maria.grigorie@umfcd.ro; 5 Department of Dialysis, Bucharest Emergency University Hospital, 050098 Bucharest, Romania; delia.timofte@gmail.com

**Keywords:** sepsis, hemodialysis, end-stage renal disease, catheter-related infections, antimicrobial therapy, dialysis-associated infections, immune dysfunction, infection control

## Abstract

**Background:** Sepsis is a leading cause of morbidity and mortality among patients receiving maintenance hemodialysis (HD), reflecting a unique combination of immunologic dysfunction, comorbidities, and healthcare-related exposures. Despite advances in dialysis technology and infection control, outcomes for septic HD patients remain disproportionately poor. **Objective:** This review aims to synthesize current evidence on the epidemiology, risk factors, diagnostic challenges, and treatment considerations of sepsis in HD patients, highlighting persistent vulnerabilities and areas for clinical improvement. **Methods:** A structured narrative review was conducted, focusing on high-quality cohort studies, surveillance data, and pharmacologic analyses published over the past two decades. The literature search was performed using PubMed, Web of Science, and Google Scholar. A total of 37 studies were included in the final synthesis. Key themes were organized around epidemiologic trends, infection sources, risk modifiers, treatment outcomes, and antimicrobial considerations in the dialysis population. **Results:** The review found that sepsis in HD patients is multifactorial and systemic. Diabetes, advanced age, and central venous catheters remain strong risk factors, while a substantial proportion of infections arise from non-access-related sources. Mortality rates remain high, often due to delays in recognition, inappropriate empiric therapy, and challenges in antimicrobial dosing. Pharmacokinetic alterations in renal replacement therapy complicate treatment, requiring individualized approaches. Despite variations in infection rates across centers, systemic vulnerabilities—rather than dialysis modality alone—drive outcomes. **Conclusions:** Sepsis in hemodialysis patients is not solely a hardware-related complication but reflects deeper systemic and immunologic challenges. Improving outcomes will require earlier recognition, tailored antimicrobial strategies, standardized infection control protocols, and broader attention to patient-specific risk factors. Future research should focus on ESRD-adapted sepsis diagnostics and interventional models to reduce infection-related mortality in this high-risk group.

## 1. Introduction

Sepsis remains a critical global health issue, representing one of the leading causes of morbidity and mortality in intensive care units worldwide. According to the World Health Organization’s global epidemiological report, sepsis affects nearly 50 million individuals annually and accounts for approximately 11 million deaths—about 20% of all global deaths each year [1]. Among the populations at elevated risk, patients with end-stage renal disease (ESRD) on hemodialysis are particularly susceptible due to their immunosuppressed state, chronic exposure to invasive procedures, and frequent use of vascular access devices. Infections are the second leading cause of death in this group, surpassed only by cardiovascular disease, and often present with nonspecific or attenuated clinical signs, which may delay diagnosis and treatment [2]. Recent data indicate that approximately 30% of patients undergoing hemodialysis develop sepsis, with incidence rates exceeding 12 cases per 100 person-years—particularly among those with central venous catheters [3]. Clinical observations have shown that the presentation of sepsis in this population is frequently atypical—characterized by diminished fever response, lower leukocyte count elevations, and increased diagnostic uncertainty, necessitating careful clinical judgment and early suspicion for timely intervention [2].

These atypical clinical manifestations are not incidental but stem from the immunological and physiological disturbances inherent to end-stage renal disease, which significantly alter host defense mechanisms and sepsis pathophysiology in hemodialysis patients. Uremia and chronic systemic inflammation impair both innate and adaptive immune responses, resulting in reduced neutrophil chemotaxis, diminished monocyte function, and impaired antigen presentation, all of which weaken the host’s ability to mount an effective defense against pathogens [4]. In addition, the frequent use of vascular access devices such as arteriovenous (AV) shunts and central venous catheters predisposes patients to recurrent bloodstream infections and biofilm-mediated seeding, which can rapidly escalate into septic shock [5]. Compounding these risks are diagnostic limitations: ESRD patients often exhibit an attenuated febrile response, baseline leukocyte abnormalities, and uremia-induced alterations in inflammatory markers such as C-reactive protein and procalcitonin, which may obscure early sepsis detection [4,6]. These combined factors not only increase the risk of delayed diagnosis and suboptimal treatment but also contribute to significantly higher rates of intensive care utilization, mechanical ventilation, vasopressor use, and in-hospital mortality in septic hemodialysis patients compared to the general population [4].

Vascular access infections remain among the most frequent and severe complications in patients undergoing hemodialysis, contributing significantly to hospitalization rates, morbidity, and mortality [7,8]. Catheter-related bloodstream infections (CRBSIs) account for more than two-thirds of access-related infections and are frequently associated with the need for catheter removal, which can jeopardize future vascular access and increase long-term reliance on central venous catheters [7,8]. The use of tunneled cuffed catheters, while often necessary, carries a two- to threefold higher risk of severe infectious complications compared to arteriovenous fistulas or grafts [8,9]. Gram-positive bacteria—particularly *Staphylococcus aureus*, including methicillin-resistant strains (*MRSA*), and *coagulase-negative staphylococci*—are the predominant pathogens in these infections [9,10]. Their presence is linked to extended antimicrobial therapy, increased hospitalization duration, and a higher likelihood of clinical deterioration [11,12]. Clinical outcomes are further compromised in patients with systemic vulnerabilities such as malnutrition, immobility, and functional decline, which heighten the risk of progression to sepsis [13].

Despite the growing awareness of infection-related morbidity and mortality in hemodialysis patients, significant gaps persist in the clinical approach to sepsis within this population. The literature remains limited regarding the diagnostic and therapeutic nuances specific to end-stage renal disease, particularly in relation to atypical clinical presentations, altered biomarker kinetics, and challenges in managing vascular access-related infections [14,15]. Furthermore, the emergence of antimicrobial-resistant pathogens such as methicillin-resistant *Staphylococcus aureus* [16], along with the limitations of conventional diagnostic methods [15], underscore the necessity for updated and comprehensive evaluations of current practices. In this context, the present narrative review synthesizes recent evidence on the diagnostic and therapeutic particularities of sepsis in patients receiving hemodialysis. It places particular emphasis on diagnostic limitations, microbial profiles, empirical and targeted antimicrobial therapies, and vascular access-related complications. By addressing key areas of uncertainty and proposing strategies for individualized care, this review aims to support more accurate risk stratification and informed clinical decision-making in a population at elevated risk for sepsis-related morbidity and mortality.

## 2. Materials and Methods

This review was conducted following a narrative review methodology. A targeted literature search was performed using PubMed, Web of Science and Google Scholar, supplemented by manual screening of reference lists from eligible articles. The literature search covered the period from January 2015 to July 2025. The final search was conducted on 22 July 2015. The search aimed to identify studies focusing on diagnostic and therapeutic challenges of sepsis in patients undergoing hemodialysis.

The following keywords and Boolean combinations were used: “hemodialysis”, “sepsis”, “end-stage renal disease”, “vascular access infection”, “catheter-related bloodstream infection”, “biofilm”, “infection biomarkers”, “antimicrobial resistance”, and “empirical antibiotic therapy”. Additional terms were incorporated to address diagnostic markers, immune dysfunction in ESRD, and treatment protocols specific to dialysis patients.

Inclusion criteria encompassed peer-reviewed original research articles, clinical trials, retrospective cohort studies, and narrative or systematic reviews published between 2015 and 2025, written in English. Studies were selected based on their relevance to the pathophysiology, microbiology, clinical presentation, diagnostic challenges, and therapeutic approaches to sepsis in the adult hemodialysis population. There were no restrictions on study design or geographic origin, provided the content offered clinically meaningful insights.

Exclusion criteria included non-English publications, articles focusing solely on pediatric or peritoneal dialysis patients, case reports without clinical or diagnostic relevance, non-peer-reviewed abstracts or opinion pieces, and studies unrelated to the sepsis–hemodialysis interface.

The initial search yielded 168 peer-reviewed publications across PubMed, Web of Science, and Google Scholar. After screening titles and abstracts for relevance and applying inclusion/exclusion criteria, a total of 37 studies were selected for full review and synthesis. Selection was performed independently by two reviewers, with discrepancies resolved through discussion. Studies were grouped thematically into four major domains: (1) diagnostic markers and clinical presentation, (2) microbial epidemiology and vascular access, (3) empirical therapy and antimicrobial resistance, and (4) clinical outcomes. No formal meta-analysis or quality scoring tools were applied due to the narrative design of this review.

## 3. Results

### 3.1. Diagnostic Particularities in Hemodialysis Patients

The clinical presentation of sepsis in patients undergoing hemodialysis is frequently atypical and may differ substantially from that seen in the general population. In hemodialysis patients, classical signs of sepsis—such as fever, leukocytosis, and hypotension—are frequently diminished, primarily due to underlying immune dysfunction and uremia-associated inflammation [4,6].

Systemic inflammatory response syndrome (SIRS) criteria are commonly not met at the time of presentation. Instead, patients may present with nonspecific manifestations such as altered mental status, vague fatigue, or vascular access dysfunction, which may delay both diagnosis and initiation of appropriate therapy [6,10].

Given the altered immune responses and biomarker kinetics in patients with ESRD, the diagnostic evaluation of sepsis in this population poses unique challenges. Table 1 summarizes the behavior and limitations of common clinical indicators and inflammatory markers in hemodialysis patients.

PCT, which is less affected by renal clearance than CRP, may accumulate to a lesser extent in chronic inflammation, accounting for its relatively superior performance in distinguishing acute infection in ESRD patients. A meta-analysis by Tao et al. [15] demonstrated that while PCT showed better overall diagnostic performance than CRP in this population, its specificity remained suboptimal, particularly in stable dialysis patients without infection.

Moreover, multidrug-resistant infections are common, particularly in patients with central venous catheters or recent hospitalization, further complicating clinical assessment. These factors contribute to diagnostic uncertainty and highlight the need for a high index of suspicion when evaluating dialysis patients for possible sepsis [10,17].

Non-access-related infections such as pneumonia and soft tissue infections also present distinct diagnostic challenges in dialysis patients. Radiographic signs of pneumonia may be obscured by chronic pulmonary congestion or volume overload, while fever or leukocytosis may be absent due to immune dysfunction. Similarly, soft tissue infections—especially in diabetic ESRD patients—may lack classical signs such as erythema or localized pain, owing to poor perfusion and peripheral neuropathy. As such, clinicians must maintain a high index of suspicion and consider early use of adjunctive biomarkers or imaging modalities like CT or MRI when these infections are suspected [6].

Given these limitations, clinicians should adopt a multi-parametric approach—integrating serial biomarker trends, access site evaluation, and changes in mental status—to ensure timely diagnosis of sepsis in this high-risk population.

### 3.2. Vascular Access and Microbiological Profiles

Infections related to vascular access are the leading cause of sepsis in patients undergoing chronic hemodialysis, particularly in those with central venous catheters (CVCs) rather than arteriovenous fistulas (AVFs) [2,4]. CVCs create a direct conduit for microbial entry into the bloodstream and are associated with a markedly increased risk of bacteremia and septic complications. This vulnerability is amplified by the immunosuppressed state of ESRD, which compromises both innate and adaptive immune responses [2].

A multicenter cohort study from the Philippines found that 56.3% of bloodstream infections in hemodialysis patients were catheter-related, with tunneled catheters accounting for 41.4% and non-tunneled for 14.9% of cases [18]. The most frequently isolated organisms included *Staphylococcus aureus* (44.9%), methicillin-resistant *S. aureus* (*MRSA*), (23.3%), *coagulase-negative staphylococci* (15.1%), and Gram-negative bacilli such as *E. coli*, *Klebsiella* spp., and *Pseudomonas aeruginosa* (25.4%). Notably, 9.5% of the Gram-negative isolates were extended-spectrum beta-lactamase (ESBL) producers, indicating the need for empiric broad-spectrum antibiotic coverage [18].

Canadian surveillance data have reported CRBSI rates ranging from 0.5 to 5.5 episodes per 1000 catheter-days in patients on hemodialysis, with *S. aureus* and *coagulase-negative staphylococci* accounting for more than half of all isolates [19]. Prolonged catheter dwell time, multiple catheter manipulations, and recent hospitalizations were key risk factors for infection [19].

A prospective study from Nepal showed that bloodstream infections comprised 61% of catheter-related infections, while local site infections accounted for 39% [20]. The most common pathogens included *coagulase-negative staphylococci* (26.8%), *S. aureus* (24.4%), and *Klebsiella pneumoniae* (21.9%). Risk factors identified included catheter duration > 30 days, recent catheter changes, and off-label catheter use for transfusion or medication.

Despite improvements in infection prevention, data from Spain show that tunneled catheter-related bloodstream infections remain disproportionately high among hemodialysis patients, with *S. aureus* and *coagulase-negative staphylococci* continuing to dominate. Alarmingly, *MRSA* remained a stable fraction of isolates over a 13-year period [21].

Emergency-only dialysis settings carry even higher infection risks. In a U.S. cohort lacking access to scheduled outpatient dialysis, the incidence of CRBSI reached 0.84 per 1000 catheter-days, with *S. aureus* (41.6%) and *MRSA* (16.8%) as the leading pathogens. These patients also experienced higher rates of recurrent infection and prolonged hospitalization [22].

Infective endocarditis (IE) is a devastating complication of catheter-related bacteremia. A Spanish national study of 9008 IE episodes found that dialysis patients had significantly higher rates of *S. aureus* (36.1%) and coagulase-negative staphylococcal infections (19.2%) compared to non-ESRD individuals. Dialysis patients also had more comorbidities and higher mortality (33.4%) [23]. Similarly, a five-year Indian study reported poor outcomes among 15 hemodialysis patients with IE, 86% of whom had tunneled catheters and prior CRBSIs. *S. aureus* was the most commonly isolated organism, and complications included stroke and septic shock [24].

Healthcare-associated infections (HCAIs) remain prevalent among patients receiving renal replacement therapy (RRT). In a multicenter study, CRBSIs were the most common HCAI (36.8%), followed by peritonitis and pneumonia. Among 382 isolates, *S. aureus*—both methicillin-sensitive and resistant—was significantly associated with CRBSIs. Key risk factors included use of multiple vascular accesses, hyperglycemia, and elevated C-reactive protein [25].

Multidrug-resistant (MDR) organisms are also increasingly frequent. A South Korean cohort reported MDR pneumonia in 22.8% of hospitalized hemodialysis patients, with *MRSA* and *P. aeruginosa* as dominant agents. Two independent predictors—recent hospitalization and a Pneumonia Severity Index > 147—were used to develop a risk score that reliably stratified patients [26].

Colonization with vancomycin-resistant *enterococci* (*VRE*) is another growing concern. A meta-analysis of 23 studies estimated a pooled *VRE* colonization rate of 6.2% in dialysis patients, with significant progression to active infection (OR: 21.62). Key predictors included prior vancomycin exposure, recent antibiotic use, and hospitalization [27].

Finally, updated U.S. CDC surveillance shows that *MRSA* continues to account for approximately 34% of dialysis-related bloodstream infections [28]. Alfano et al. further emphasized that patients with endovascular catheters and cardiac implantable devices are at particularly high risk of recurrent and severe infections, reinforcing the importance of AVF prioritization and early device-related infection surveillance [29].

To facilitate cross-study comparison, the prevalence data and microbiological profiles from key studies on vascular access infections are summarized in Table 2.

The following Table 3, summarizes the most frequently reported pathogens associated with vascular access infections in hemodialysis patients, their linked access types, reported prevalence ranges, and antimicrobial resistance traits across global studies.

Despite geographic variability, *S. aureus* (including *MRSA*) and *coagulase-negative staphylococci* consistently dominate vascular access infections. CVCs, particularly tunneled variants, remain the highest-risk access type globally. These findings strengthen international guidelines advocating early AVF placement and surveillance protocols targeting MDR organisms in dialysis centers.

### 3.3. Empirical Therapy and Antimicrobial Resistance

Empirical antimicrobial therapy in hemodialysis patients must balance urgency with broad-spectrum efficacy, particularly given the high prevalence of bloodstream infections (BSIs) due to MDROs (Multidrug-Resistant Organisms). While Gram-positive bacteria—especially *MRSA*—remain predominant, increasing reports of resistant Gram-negative bacilli have made dual empiric coverage essential in most settings [33,34].

Surveillance data from Canadian cohorts demonstrated that an empiric regimen combining vancomycin with a Gram-negative agent (such as third-generation cephalosporins or β-lactam/β-lactamase inhibitors) achieved up to 99.7% predicted coverage for clinical isolates. In contrast, cefazolin monotherapy offered significantly lower coverage (~68%), rendering it suboptimal in high-risk populations [33]. These findings support the continued use of vancomycin-based empiric therapy, particularly in regions with elevated *MRSA* prevalence.

Given the variability in pathogen resistance and patient vulnerability, empirical antibiotic selection in hemodialysis-associated sepsis should be guided by clinical risk factors. Table 4 outlines recommended initial regimens based on stratified risk categories, coverage goals, and referenced clinical guidance.

This risk-stratified framework emphasizes the need to tailor empirical therapy to patient-specific exposures and local antibiogram trends. Inappropriate delays or monotherapy in high-risk patients—especially those with CVCs or recent hospitalization—can significantly increase mortality and length of stay.

The clinical consequences of inadequate empirical therapy are substantial. A study in Greece reported that inappropriate initial antibiotic selection was independently associated with adverse outcomes, particularly in catheter-dependent patients and those with polymicrobial infections, thus reinforcing the need for prompt, appropriately targeted empiric treatment in hemodialysis-associated sepsis [34].

In addition to appropriate agent selection, optimizing antimicrobial dosing based on the dialysis modality is essential. Patients receiving CRRT may require more frequent or higher dosing of time-dependent antibiotics, such as β-lactams, due to continuous extracorporeal clearance. In contrast, IHD often permits post-dialysis dosing with longer intervals. For instance, meropenem may be administered every 8 h during CRRT to maintain target plasma concentrations, but can be spaced to 12–24 h in IHD depending on residual renal function. Vancomycin dosing also varies significantly, with higher loading doses and frequent monitoring required in CRRT. Aminoglycosides, while effective in select cases, require caution in both modalities due to nephrotoxicity and variable clearance.

Beyond treatment considerations, antimicrobial stewardship remains a cornerstone of infection control. Routine microbiological surveillance, adherence to infection prevention protocols, and strategies to reduce CVC usage are vital in mitigating resistance trends and optimizing outcomes [35].

Emerging resistance patterns also call into question the long-term utility of antimicrobial prophylaxis, such as gentamicin-heparin antimicrobial locks (AMLs). In a New Zealand study, prolonged AML use correlated with a marked rise in gentamicin resistance among *coagulase-negative staphylococci*, increasing from 25% to 71% over three years [32]. Despite their demonstrated efficacy in reducing catheter-associated bloodstream infections (CABSIs), this resistance pattern highlights the risk of selective antimicrobial pressure and underscores the importance of continuous surveillance.

International studies further illustrate the growing burden of resistance. In Saudi Arabia, Gram-negative organisms comprised more than half of BSI isolates, and 50% of *S. aureus* isolates were methicillin-resistant. Nearly one-third of all isolates exhibited multidrug resistance, necessitating empiric coverage for both *MRSA* and resistant Gram-negatives [36]. Similarly, a Brazilian study reported high rates of methicillin resistance (71.8% of *S. aureus* isolates) and noted the presence of extended-spectrum beta-lactamase (ESBL)—producing *Klebsiella pneumoniae* and *Pseudomonas aeruginosa* with reduced susceptibility to fluoroquinolones and aminoglycosides [30].

Resistance trends also vary geographically. A retrospective Chinese study found that although *S. aureus* remained dominant (45.5%), only 20% of isolates were methicillin-resistant, and all retained sensitivity to vancomycin and linezolid. In contrast, 54.5% of Gram-negative isolates were resistant to third-generation cephalosporins, though most remained susceptible to imipenem, gentamicin, ciprofloxacin, and levofloxacin—agents that may offer effective empiric options in similar contexts [31].

Recent metagenomic sequencing in HD units has identified vascular catheter colonization by *K. pneumoniae* and *P. aeruginosa* harboring ESBL and carbapenemase genes. These findings raise concerns about biofilm-associated reservoirs of resistance not detectable through routine cultures, and support routine surveillance beyond conventional microbiology [37].

The feasibility of implementing standardized infection control protocols across diverse healthcare systems is limited by the substantial geographic variability in resistance patterns, healthcare infrastructure, and infection types. As summarized in Table 2, rates of MRSA, ESBL-producing Gram-negatives, and VRE differ significantly across regions—from high MRSA prevalence in Brazil and the USA to lower levels in China. Surveillance quality also varies, with national networks active in some countries and single-center studies in others. These discrepancies complicate the application of uniform empirical regimens or prevention strategies. While core principles such as hand hygiene and catheter care are universally applicable, effective implementation requires local epidemiologic data, flexible protocols, and resource-sensitive adaptations.

### 3.4. Clinical Outcomes and Mortality Predictors in Hemodialysis-Associated Sepsis

Hemodialysis patients with sepsis face disproportionately high morbidity and mortality. A 21-year cohort study of 453 hospitalized patients with *Staphylococcus aureus* bacteremia (SAB) showed a concerning rise in SAB-attributable mortality, persistent bacteremia, and metastatic complications—each increasing annually by up to 0.86%. Outcomes were worse in infections not related to vascular access (OR 3.20, 95% CI 1.36–7.55), especially those caused by *MRSA* USA300 strains, a virulent community-associated strain increasingly seen in dialysis-related infections (OR 2.96, 95% CI 1.12–7.83) [38].

These findings are echoed in a Japanese population-based study, which reported a 90-day fatality rate of 6% among 200 patients with hemodialysis-associated infection (HAI). Mortality was significantly higher in patients with severe comorbidities (HR 1.87, 95% CI 1.11–3.14) and in those treated at smaller outpatient clinics. Notably, variation in facility practices emerged as a dominant determinant of infection-related outcomes [39].

Further emphasizing the systemic risk, CDC surveillance data from over 6400 outpatient dialysis facilities in the U.S. documented ~37,000 bloodstream infections and over 80,000 infection events in 2014. CVCs were a major risk factor, with infection rates nearly tenfold higher than with AVFs. Alarmingly, 70% of bloodstream infections required hospitalization, and 17% progressed to sepsis or septic shock, with a 30-day mortality of 20% [29].

Hospital-based studies further corroborate this burden. In a 5-year cohort of 234 hemodialysis patients with bloodstream infections, the 30-day mortality was 16.2%. Predictors included ICU admission (OR 7.2), infection with MDR organisms (OR 4.6), and delayed source control (OR 3.9) [40]. MDR organisms were identified in 24% of isolates and were linked to longer hospitalizations and higher recurrence. Notably, over a quarter of patients received inappropriate empiric therapy, which was associated with increased mortality.

This pattern extends to dialysis patients presenting with septic shock. A cohort of 137 ESRD patients showed a hospital mortality rate of 20%, with age, low systolic blood pressure, and elevated lactate as independent risk factors. Externalized CVCs were more prevalent among nonsurvivors, while AV access was protective. Despite adherence to antibiotic guidelines, fewer than 40% received adequate fluid resuscitation, raising concerns about treatment adequacy in this population [41].

Across studies, mortality in hemodialysis-associated sepsis consistently exceeds that of the general population. Vascular access type, comorbid burden, and timely source control emerge as key modifiable risk factors. These results underscore the importance of stratified risk evaluation, timely hemodynamic support, and context-specific antimicrobial selection to reduce morbidity and mortality.

## 4. Discussion

The burden of sepsis in HD patients remains alarmingly high, despite decades of advances in dialysis technology, infection surveillance, and antimicrobial therapy. Our review reinforces previous findings and highlights a persistent vulnerability to infection that transcends dialysis vintage, modality, and geographic setting. This systemic risk profile, as previously documented by Lafrance et al. [42], continues to manifest through rising sepsis-related hospitalizations and a disproportionate share of infection-related mortality in the ESRD population.

Historical data from Powe et al. [43] and Jaar et al. [44] already identified CVCs, diabetes, and advanced age as key risk factors—findings that remain consistent in more recent analyses. Our synthesis reveals a striking constancy in the risk landscape over the past two decades, suggesting that although vascular access strategies have evolved, the immunologic and metabolic milieu of ESRD continues to predispose patients to severe infections and poor outcomes.

Infections unrelated to vascular access—such as pneumonia, soft tissue infections, and limb ulcers—represent a major but underrecognized burden in dialysis patients. Berman et al. [45] observed that vascular access accounted for only a minority of infectious episodes, reinforcing the need to broaden diagnostic suspicion beyond catheter sites. Lower respiratory tract infections, soft tissue infections, and limb ulcers represent significant infection sources, often community-acquired but colonized with nosocomial pathogens due to repeated antibiotic exposure and outpatient cohorting. This convergence of community and healthcare-associated microbial profiles complicates both diagnosis and empiric treatment strategies in HD patients.

Diabetes mellitus emerged as a recurrent and powerful driver of sepsis risk, consistent with Jaar et al.’s observations [44]. Our synthesis reaffirms that diabetic HD patients experience more frequent and severe septic episodes and exhibit higher sepsis-related mortality, underscoring the need for personalized risk stratification, vigilant surveillance, and aggressive early management.

Mortality trends in dialysis-associated sepsis remain sobering. The binational cohort data from Chong et al. [46] showed infection-related deaths still occur at rates more than 20 times higher than in the general population, despite a declining standardized mortality ratio (SMR) over recent decades. These findings are paralleled by the landmark study by Sarnak and Jaber [47], which emphasized systemic immune dysfunction rather than vascular access alone as the primary driver of infection-related mortality. The evidence indicates that mortality is shaped by multifactorial vulnerabilities—metabolic derangements, malnutrition, chronic inflammation, and delayed recognition—rather than access type alone.

The critical role of empiric antimicrobial therapy cannot be overstated. Clark et al. [48] demonstrated that dialysis patients frequently receive delayed or suboptimal empiric therapy, in part due to diagnostic uncertainty and pharmacokinetic complexities. These shortcomings are magnified in peritoneal dialysis (PD) populations, where mortality from sepsis is notably higher. As outlined by Hoff et al. [49], the pharmacodynamics of antibiotics are profoundly altered by renal replacement modalities, necessitating individualized dosing and therapeutic drug monitoring—practices that remain inconsistently applied across institutions.

In our synthesis, several studies including that of AlQahtani et al. [50] and the U.S. CDC surveillance [29] showed that CVC-related bloodstream infections account for the majority of infectious events in HD patients. Yet, considerable inter-facility variability exists in both infection rates and antimicrobial prescribing patterns. This inconsistency highlights a gap between surveillance data and actionable prevention protocols. This pattern highlights not only the minimization of CVC use but also the standardization of infection control measures and empiric therapy algorithms tailored to regional microbiologic trends.

The comparative summary table of vascular access infections further illustrates this variability, showing CRBSI incidence rates ranging from 0.84 per 1000 catheter-days in U.S. emergency-only dialysis patients [22] to nearly 7 per 1000 catheter-days in Nepal and the Philippines [18,20]. While *S. aureus* and coagulase-negative staphylococci were consistently dominant pathogens across all regions [18,19,21,22,29,30,31], notable geographic differences emerged. Gram-negative organisms, including ESBL-producing Enterobacteriaceae and multidrug-resistant non-fermenters, accounted for over half of infections in Asian cohorts [18,20,31], whereas Gram-positive organisms predominated in North American and European surveillance data [19,21,29]. Catheter dependence was repeatedly identified as the strongest risk factor [2,18,19,24,29,30], but other contributors such as hospitalization, prior antibiotic exposure, and socioeconomic disparities also played significant roles [25,29,30].

Longitudinal immune dysfunction, as described by Habas et al. [51], further complicates sepsis recognition and management. Neutrophil dysfunction, malnutrition–inflammation complex syndrome (MICS), and anemia reduce infection resilience and therapeutic response. These immunologic impairments, often intrinsic to ESRD, perpetuate high sepsis mortality even in patients receiving timely and guideline-concordant therapy.

**Figure 1:**
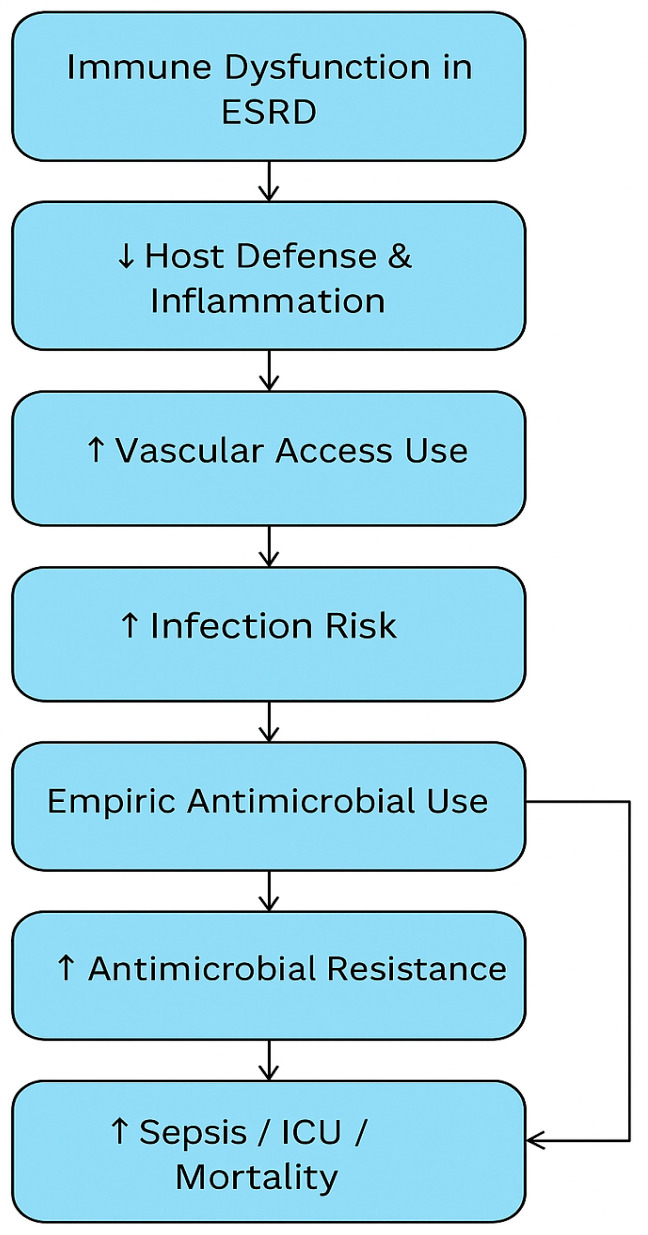
Conceptual framework illustrating the interplay between immune dysfunction, vascular access, antimicrobial exposure, antimicrobial resistance, and adverse clinical outcomes in patients with ESRD undergoing hemodialysis.

Ultimately, our synthesis reveals that the infection burden in HD patients is not simply a reflection of vascular access type or dialysis technique—it is the cumulative result of immune suppression, systemic inflammation, delayed recognition, and evolving antimicrobial resistance. Future interventions must therefore move beyond technical access improvements to embrace holistic risk mitigation, including early diagnostic markers adapted to ESRD physiology, personalized antimicrobial strategies, and rigorous implementation of infection prevention frameworks across dialysis networks. To illustrate these interrelated pathways, we present a conceptual framework (Figure 1) that links immune dysfunction, vascular access, antimicrobial resistance, and clinical outcomes in hemodialysis patients.

Currently, there are no universally accepted diagnostic criteria tailored specifically to the physiologic and immunologic characteristics of ESRD patients. Existing definitions, such as Sepsis-3, rely on inflammatory and hemodynamic parameters that may be atypical or attenuated in this population, contributing to diagnostic delays and underrecognition [4,6,14]. Moreover, validation of conventional infection biomarkers remains limited in this group. Although procalcitonin may perform better than CRP, its specificity remains suboptimal, particularly in clinically stable dialysis patients [15]. These limitations represent key knowledge gaps and should be prioritized in future research aimed at improving sepsis detection and outcomes in ESRD populations.

Looking forward, several areas warrant targeted investigation. First, there is a critical need to develop and validate sepsis biomarkers specific to the ESRD population, given the limitations of conventional indicators such as CRP and procalcitonin in this group. Second, prospective trials assessing antimicrobial pharmacokinetics and optimal dosing regimens across dialysis modalities—including IHD, PIRRT, and CRRT—are essential to improve time-to-therapeutic efficacy. Third, comparative studies examining infection prevention practices across dialysis centers may help identify scalable models for standardization and improvement. Finally, future research should focus on non-access-related infection pathways, such as pulmonary or cutaneous sources, which remain underrecognized yet clinically impactful. Together, these priorities could form the basis for a more tailored, preemptive, and outcome-oriented approach to sepsis care in dialysis patients.

## 5. Conclusions

Sepsis continues to pose a severe and unresolved threat to patients receiving maintenance hemodialysis, driven by a multifactorial interplay of immune dysfunction, metabolic comorbidities, and prolonged healthcare exposure. While advances in dialysis techniques and infection surveillance have occurred, they have not translated into meaningful reductions in sepsis-related mortality.

Our review highlights the persistent nature of this risk, with little change in septic complication profiles over the past two decades. The frequent involvement of multidrug-resistant organisms, coupled with atypical clinical presentations, necessitates early recognition and empirically appropriate, renal-adjusted antimicrobial therapy.

The limited progress in improving outcomes reflects not only diagnostic and therapeutic complexity, but also systemic deficiencies in infection prevention, individualized risk stratification, and inter-institutional care variability. Addressing these gaps requires a paradigm shift—toward earlier detection, non-access-related infection vigilance, and context-specific antimicrobial protocols.

Moving forward, a paradigm shift is needed—one that emphasizes early detection, tailored antimicrobial regimens, and broader consideration of non-access-related infection sources. By prioritizing both patient-specific vulnerabilities and system-level interventions, we can begin to close the persistent outcome gap in septic dialysis patients.

## 6. Strengths and Limitations

This review has several limitations. First, it was conducted as a narrative rather than a systematic review, without the use of formal quality assessment tools. As such, it may be more vulnerable to selection bias and does not allow for meta-analysis. Second, the included studies were heterogeneous in design, population, and outcome measures, limiting the comparability of findings. Third, most of the available evidence originates from high-income countries, which may reduce the generalizability of our synthesis to resource-limited settings where dialysis practices and antimicrobial resistance patterns differ. Finally, as with all reviews, publication bias remains a concern, as negative or confirmatory findings are less likely to be published.

Despite these limitations, the review also has important strengths. We conducted a broad multi-database search, supplemented by manual reference screening, which ensured a comprehensive capture of relevant studies. The synthesis integrates data across epidemiology, microbiology, diagnostics, and therapeutics, providing a multidimensional overview of sepsis in hemodialysis patients. By structuring the discussion around key clinical domains, the review highlights both current challenges and emerging opportunities, offering practical insights for clinicians while also outlining priorities for future research.

## Figures and Tables

**Table 1:** Diagnostic Challenges and Limitations of Sepsis Markers in Hemodialysis Patients.

Clinical Feature/Marker	Behavior in Hemodialysis Patients	Diagnostic Limitation	Clinical Interpretation
Fever	Often absent or attenuated	Reduced sensitivity as early marker	Use in combination with other markers and clinical signs
Leukocytosis	Blunted due to baseline immune dysregulation	Difficult to distinguish from chronic baseline elevations	Interpret in context of patient’s baseline values
Hypotension	May appear late or in severe stages	Non-specific in ESRD population	Requires high suspicion in absence of classical signs
Altered Mental Status	Frequently observed as an early presentation	Nonspecific; may delay recognition of sepsis	Warrants early diagnostic workup, especially if vascular access dysfunction is also present
C-Reactive Protein (CRP)	Chronically elevated, nonspecific	Low specificity due to uremia and systemic inflammation	Trending values may help monitor response to therapy
Procalcitonin (PCT)	Elevated even without infection; better sensitivity than CRP	Improved performance vs. CRP but still lacks specificity in stable patients	Supports infection suspicion when rising acutely in symptomatic patients

**Table 2:** Comparative Summary Table of Vascular Access and Related Infections in Hemodialysis Patients.

Country/Region	Study Design	Sample Size	Prevalence/Incidence	Predominant Pathogens	Key Findings
Lebanon [2]	Single-center retrospective	90	26.7% catheter infections; 39/90 bacteremic	*E. coli* 24.4%, *CoNS* 22.2%, *Klebsiella* 7.8%, *Pseudomonas* 7.8%, *Enterococcus* 6.7%, *S. aureus* 5.6%	Catheter infections leading cause of bacteremia; in-hospital mortality 26.7%
Saudi Arabia [4]	Retrospective ICU cohort	8803 (730 ESRD)	8.3% ESRD among sepsis admissions; hospital mortality 49% vs. 32%	Not reported	Higher mortality in ESRD sepsis; OR 1.44; predictors = ventilation, liver disease, vasopressors
Philippines [18]	Retrospective cohort	707 (197 CRBSI)	CRBSI 6.72/1000 catheter-days; relapse 5.08%; reinfection 15.7%; mortality 6.1%	Gram-negatives 52% (*Burkholderia* 13%, *Enterobacter* 13%, *Acinetobacter* 11%); *CoNS* 34.5%, *S. aureus* 13%	44.5% MDROs; risk factors = autoimmune disease, frequent CVC use; right-sided access protective
Canada [19]	Narrative review (cohorts and surveillance)	Multiple (e.g., SPIN-HD, n = 527, 94)	CRBSI 1.2–2.5/1000 pt-days; SPIN-HD 3.7/1000 procedures	*S. aureus* 32–55%, *CoNS* 14–40%, *Enterococcus* 5–7%, *Pseudomonas* 2–3%, *Klebsiella* 1–4%, *Candida* 1–3%	High catheter use (49%); MRSA major concern; prevention strategies emphasized
Nepal [20]	Prospective cohort	594 insertions (41 CRI)	CRI 6.94/1000 catheter-days; 61% CRBSI, 39% CRLI	*CoNS* 26.8%, *S. aureus* 24.4%, *Klebsiella* 21.9%, *Proteus* 9.7%, *E. coli* 7.3%, *Pseudomonas* 7.3%	Risk factors: prolonged catheter >30 days, recent CVC change, IV med use
Spain [21]	Prospective multicenter surveillance	9290 CRBSI episodes	Decline from 0.29 to 0.13/1000 pt-days; 62.7% CVC	*CoNS* 39.5%, *S. aureus* 24.6%, *Enterobacteriaceae* 18.4%, *Candida* 5.9%, *Pseudomonas* 5.2%	Shift from *CoNS* to *S. aureus* dominance; PVC/PICVC infections rising
USA (Houston) [22]	Retrospective cohort	329 emergency-only HD	CRBSI 0.84/1000 catheter-days; 17% recurrent	*MSSA* 24.8%, *MRSA* 16.8%, *CoNS* 13.9%, *Enterococcus* 5%, *Enterobacter* 16.8%, *Klebsiella* 4%	High recurrence; 4% mortality; prolonged hospital stay
India [24]	Retrospective cohort	15 HD with IE	86% tunneled catheters; 66% prior CRBSI	*S. aureus* 46%, 3 fungal IE	Complications: shock, stroke, embolism; mortality 53%
Malaysia [25]	Multicenter retrospective	400 ESRD on RRT	HCAI prevalence 43.5%; CRBSI 36.8%; peritonitis 25.8%; pneumonia 21.2%	53.4% Gram-positive, 42.4% Gram-negative; *MSSA* and *MRSA* linked with CRBSI	Risk factors: multiple accesses, hyperglycemia, hyponatremia, high CRP
South Korea [26]	Multicenter retrospective cohort	105 HD pneumonia cases	22.8% MDR pneumonia; mortality 7.6% overall, 25% MDR vs. 2.4% non-MDR	*S. aureus* 16.1% (*MRSA* 9.5%), Klebsiella 10.4%, *S. pneumoniae* 9.5%, *Pseudomonas* 6.6%, *Acinetobacter* 5.7%	Predictors: recent hospitalization, PSI >147; risk stratification model proposed
Multinational [27]	Systematic review and meta-analysis	4842 patients, 23 studies	Pooled VRE colonization 6.2%; North America 5.2%	*VRE*	Risk factors: antibiotics, vancomycin, hospitalization; colonization ↑ risk of infection (OR 21.62)
USA [29]	National surveillance (NHSN + EIP)	4840 facilities; 14,822 BSIs	*S. aureus* BSI 4248/100,000 person-years; 34% of BSIs; 38% MRSA	*S. aureus* (*MSSA* + *MRSA*)	CVC strongest risk factor (6× vs. AVF); disparities by race/SES
Brazil [30]	Retrospective case–control	162 (81 BSI cases, 81 controls)	BSIs 100% (by design); Gram+ 72.8%, Gram− 25.9%, fungi 1.2%	*S. aureus* 32% (39% *MRSA*), *S. epidermidis* 13.6% (100% MR), Enterococcus 3.7% (67% VRE)	Risk factors: CVC use OR 11.2, hospitalization OR 6.6, antibiotics OR 2.5; mortality 18.5%
China [31]	Retrospective observational	75 TCC with CRBSI (33 positive cultures)	Blood culture positivity 44%; Gram+ 66.7%, Gram− 33.3%	*S. aureus* 45.5% (20% *MRSA*), *S. epidermidis* 9.1%, *Enterococcus* 6.1%, *Klebsiella* 6.1%, *Enterobacter* 6.1%	High resistance: GP 100% penicillin-R; GN > 50% resistant to ceftriaxone; all sensitive to carbapenems and fluoroquinolones

**Table 3:** Common Pathogens and Their Associations with Hemodialysis Vascular Access Types.

Pathogen	Associated Access Type	Reported Prevalence (%)	Antimicrobial Resistance Traits
***Staphylococcus aureus* (MSSA/*MRSA*)**	Central venous catheters (CVC), especially tunneled	20–45% overall; up to 23% *MRSA* [18,19]	Methicillin resistance (*MRSA*), biofilm formation [19,21]
***Coagulase-negative staphylococci***	CVCs and prosthetic grafts	15–30% [18,19,21]	Gentamicin resistance with AML use, biofilms [32]
***Klebsiella pneumoniae* (ESBL-producing)**	CVCs; prolonged catheter use	9–22% [18,20,21]	ESBL enzymes, reduced fluoroquinolone efficacy [18,20,30]
***Escherichia coli***	Non-tunneled catheters, emergency access	8–15% [18,20]	Potential for ESBL or carbapenem resistance [20,31]
***Pseudomonas aeruginosa***	Multiple catheter manipulations; hospital exposure	7–14% [18,21,26]	Multidrug resistance, efflux pumps, β-lactamases [26,30]
***Vancomycin-resistant Enterococci* (*VRE*)**	Recent hospitalization; prior vancomycin use	6–10% colonization; high risk of infection [27]	Intrinsic vancomycin resistance [27]

**Table 4:** Empirical Antimicrobial Strategies for Hemodialysis Patients with Suspected Sepsis.

Risk Category	Empirical Antibiotic Regimen	Coverage Goals	Notes / References
**Low Risk** (Stable patient, AVF access, no recent hospitalization)	Cefazolin ± aminoglycoside or fluoroquinolone	Gram-positive coverage; Gram-negative if signs of systemic infection	Appropriate in low MDRO settings [33]
**Moderate Risk** (Recent outpatient infection, prosthetic access)	Vancomycin + third-generation cephalosporin (e.g., ceftriaxone)	*MRSA* and Gram-negative organisms; broader spectrum needed	Consider hospital antibiogram data [33,34]
**High Risk** (CVC, recent hospitalization or prior MDRO)	Vancomycin + β-lactam/β-lactamase inhibitor (e.g., piperacillin-tazobactam)	Broad Gram-positive + Gram-negative including ESBLs and *MRSA*	Delay in appropriate therapy linked to mortality [34]
**Severe Risk** (Septic shock, MDR colonization or ICU admission)	Vancomycin + carbapenem (e.g., meropenem) ± aminoglycoside	Coverage for *MRSA*, *ESBL*, *Pseudomonas*, and potential carbapenem-resistant organisms	Requires urgent escalation; consider stewardship input [32,33,34,35]

## Data Availability

No new data were created or analyzed in this study. Data sharing is not applicable to this article.

## Abbreviations

The following abbreviations are used in this manuscript:

AML	Antimicrobial Lock
AV	Arteriovenous
AVF	Arteriovenous Fistula
BSI	Bloodstream Infection
CABSI	Catheter-Associated Bloodstream Infection
CDC	Centers for Disease Control and Prevention
CRBSI	Catheter-Related Bloodstream Infection
CRP	C-Reactive Protein
CRRT	Continuous Renal Replacement Therapy
CVC	Central Venous Catheter
ESBL	Extended-Spectrum Beta-Lactamase
ESRD	End-Stage Renal Disease
HAI	Hemodialysis-Associated Infection
HCAI	Healthcare-Associated Infection
IE	Infective Endocarditis
IHD	Intermittent Hemodialysis
MDR	Multidrug-Resistant
MDRO	Multidrug-Resistant Organism
MICS	Malnutrition–Inflammation Complex Syndrome
MRSA	Methicillin-Resistant Staphylococcus aureus
MSSA	Methicillin-Sensitive Staphylococcus aureus
OR	Odds Ratio
PCT	Procalcitonin
PD	Peritoneal Dialysis
PIRRT	Prolonged Intermittent Renal Replacement Therapy
RRT	Renal Replacement Therapy
SAB	Staphylococcus aureus Bacteremia
SIRS	Systemic Inflammatory Response Syndrome
SMR	Standardized Mortality Ratio
VRE	Vancomycin-Resistant Enterococci
